# Supplementary material for: Molecular and Cellular Risk Assessment of Healthy Human Cells and Cancer Human Cells Exposed to Nanoparticles
Source: Int J Mol Sci. 2019 Dec 28;21(1):230. doi: 10.3390/ijms21010230 (PMC6981945; doi:10.3390/ijms21010230)
Supplement: Supplementary file 1 [file ijms-21-00230-s001.pdf]

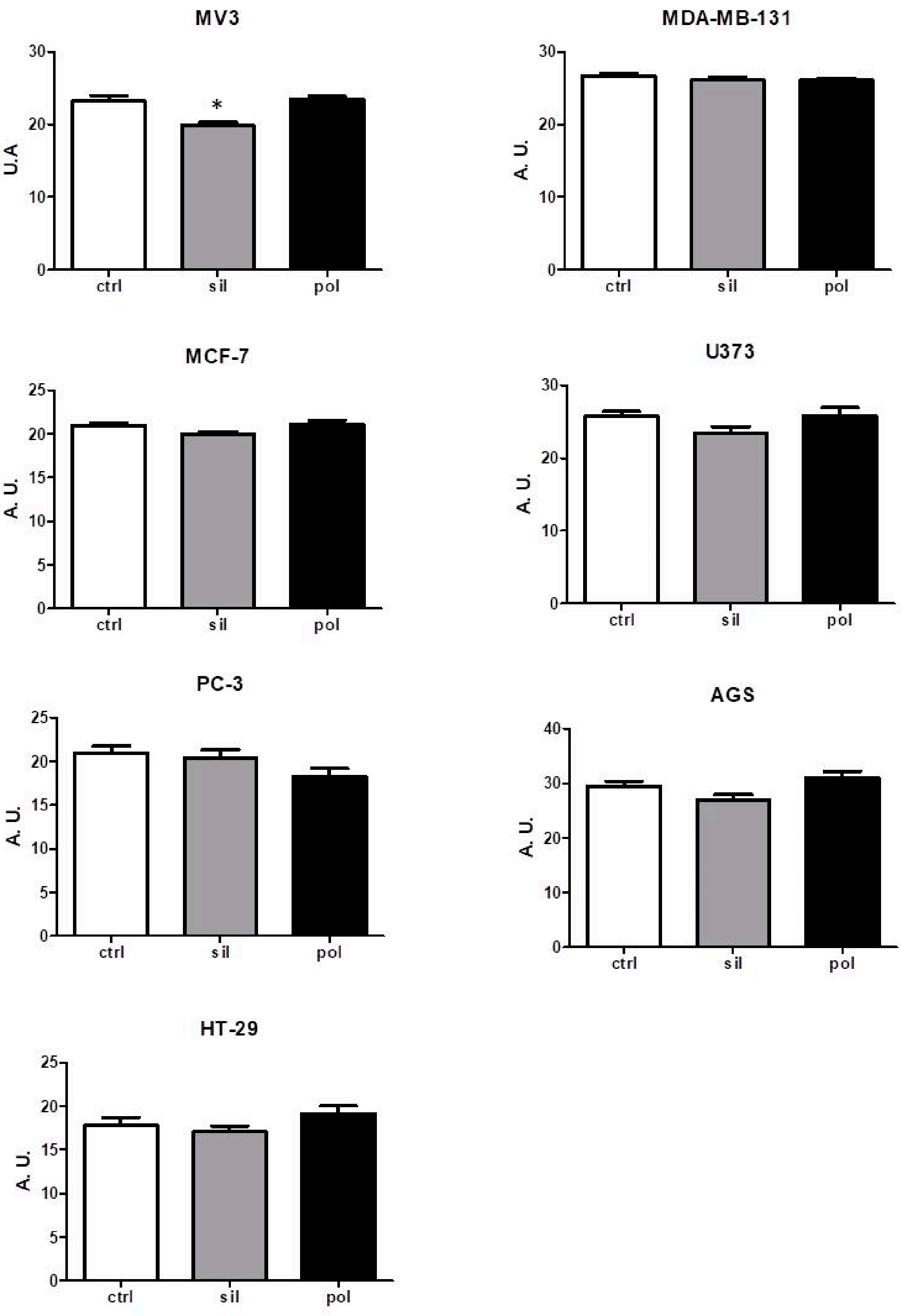

MV3
U.A
30
20
10
0
*
ctrl
sil
pol
MDA-MB-131
A. U.
30
20
10
0
ctrl
sil
pol
MCF-7
A. U.
25
20
15
10
5
0
ctrl
sil
pol
U373
A. U.
30
20
10
0
ctrl
sil
pol
PC-3
A. U.
25
20
15
10
5
0
ctrl
sil
pol
AGS
A. U.
40
30
20
10
0
ctrl
sil
pol
HT-29
A. U.
25
20
15
10
5
0
ctrl
sil
pol

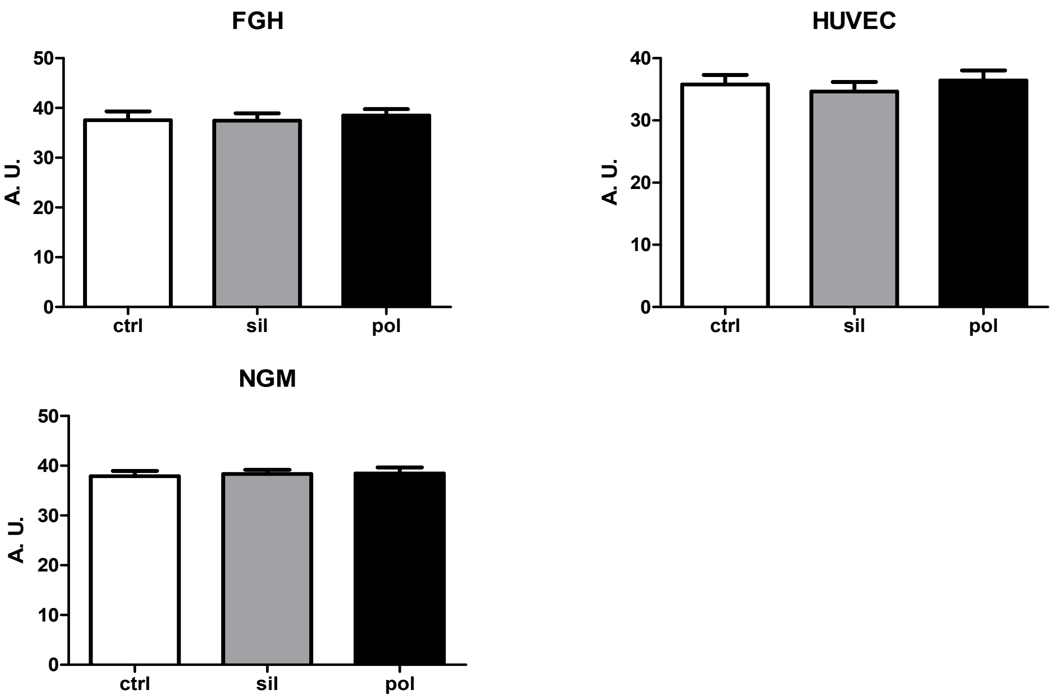

Figure S1. Effects of nanoparticles in tumor and non-tumor cells actin cytoskeleton. Cells were incubated with polymeric (pol) or silica (sil) nanoparticles (20µg/mL) for 24hs. Filamentous actin was labeled with TRITC-phalloidin and nuclei were stained with DAPI. Representative graphics showing the relative fluorescence intensity of TRITC-phalloidin from 3 individual experiments captured at 400x magnification *$p<0.05$.

Supplementary methodology: fluorescence **quantification analyses**

Cells (7 x 10^4 cells/well) were cultured on sterile glass coverslipsthen for 24 hours. Then, cells were fixed with 4% paraformaldehyde in sucrose/PBS solution for 20 min at room temperature, permeabilized with Triton X-100 (0.1%)/PBS for 5 min and blocked with 5% BSA for 30 minutes. Then, cells were labeled with TRITC-phalloidin (1:400) overnight at 4°C. Finally, slides were mounted using ProLong Gold antifade reagent with 4,6-diamidino-2-phenylindole (DAPI) for nuclear staining. Coverslips were examined under an Olympus BX40 microscope equipped for epifluorescence at 400x magnification. Fluorescence intensity was measured on Icy bioimage analysis version 1.9.5.1 and Excel. The result shows the mean ± SD of the relative fluorescence intensity.
